# Classical molecular dynamics investigations of biphenyl-based carbon nanomembranes

**DOI:** 10.3762/bjnano.5.98

**Published:** 2014-06-17

**Authors:** Andreas Mrugalla, Jürgen Schnack

**Affiliations:** 1 Fakultät für Physik, Universität Bielefeld, Postfach 100131, D-33501 Bielefeld, Germany

**Keywords:** biphenyls, carbon nanomembranes, classical molecular dynamics

## Abstract

**Background:** Free-standing carbon nanomembranes (CNM) with molecular thickness and macroscopic size are fascinating objects both for fundamental reasons and for applications in nanotechnology. Although being made from simple and identical precursors their internal structure is not fully known and hard to simulate due to the large system size that is necessary to draw definite conclusions.

**Results:** We performed large-scale classical molecular dynamics investigations of biphenyl-based carbon nanomembranes. We show that one-dimensional graphene-like stripes constitute a highly symmetric quasi one-dimensional energetically favorable ground state. This state does not cross-link. Instead cross-linked structures are formed from highly excited precursors with a sufficient amount of broken phenyls.

**Conclusion:** The internal structure of the CNM is very likely described by a disordered metastable state which is formed in the energetic initial process of electron irradiation and depends on the process of relaxation into the sheet phase.

## Introduction

Freestanding carbon nanomembranes are produced from molecular precursors such as biphenylthiols (BPT). The precursors self-assemble in monolayers on gold surfaces and are then polymerized by irradiation with electrons [1–3]. The product is a membrane, whose thickness, homogeneity and surface chemistry are related to the molecular precursor. So far several classes of precursors have been exploited [3]. One of the major unsolved questions is the internal structure of these membranes, since the structure cannot be determined by X-ray diffraction. In [4] quantum chemical calculations were performed for various dimers of biphenyls, which left open how the precursor molecules interlink laterally. A first small-scale quantum calculation (using ARGUS Lab) of a two-dimensional cutout of 6 by 5 biphenyls is reported in [5]. These calculations suggest that the regular structure of the precursor self-assembled monolayer (SAM) turns into a disordered sheet. Nevertheless, the simulations of the very small system do not allow definite conclusions about the structure of the extended sheet. On the other hand, the quantum mechanical simulation of extended systems even by means of density functional theory (DFT) has to assume a regular lattice and can treat only small unit cells [6–8]. Consequently, the resulting structure is also regular [8]. If one, as in the present case, can expect that the structure is irregular, i.e., a lattice structure as in solids cannot be assumed, a quantum mechanical simulation is virtually impossible.

In this article we therefore resort to classical molecular dynamics simulations, which allow to simulate up to several millions of carbon atoms. In order to account for the very flexible sp*^n^*-binding modes of carbon we use the modern carbon–carbon potential of Nigel Marks [9], which has been demonstrated to be able to simulate extended carbon structures [10–11]. We focus our investigations on CNMs made from biphenylthiols. The simulation results of our energy minimizing procedure yield – depending on the initial state – a large variety of structures, among which a very regular one made of parallel graphene stripes has the lowest energy. Our hypothesis is, that in a realistic synthesis process such an idealized state is not reached, instead the system “freezes” into a metastable irregular configuration that is laterally linked through carbon bonds of broken phenyls. We show that such structures indeed form in our simulations. The article is organized as follows. In the next two sections we shortly repeat the essentials of our classical molecular dynamics simulations. The main section discusses the results and our interpretation. The article closes with an outlook.

## Classical carbon–carbon interaction

**Figure 1:**
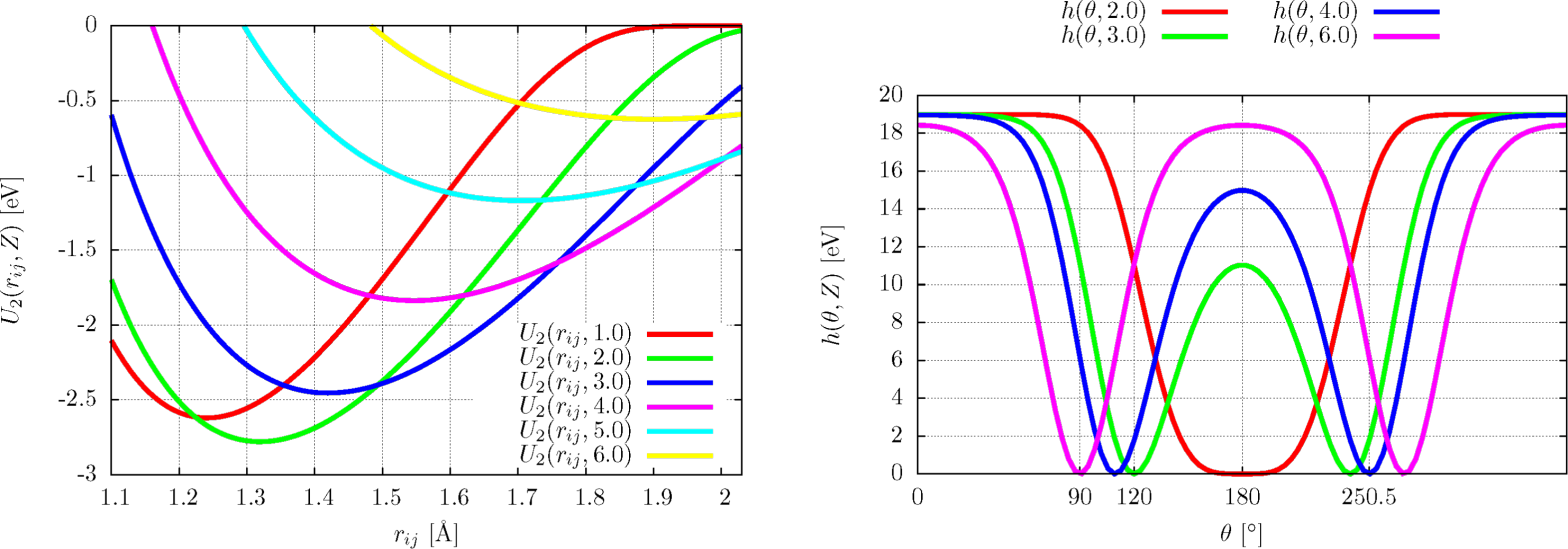
Pictorial representation of the major ingredients of the carbon–carbon potential in Equation 1, compare also [9]. L.h.s.: radial dependence of the two-body potential for various coordination numbers. R.h.s.: coordination number dependence of angular-dependent part of the three-body potential.

A realistic classical carbon–carbon interaction must be able to account for the various sp*^n^*-binding modes. Two potentials, developed by Tersoff and Brenner, have been used for carbon materials as well as for hydro-carbons [12–14]. In our investigations we employ the improved potential by Marks [9]. This potential comprises density-dependent two- and three-body potentials, *U*_2_ and *U*_3_ respectively,

[1]
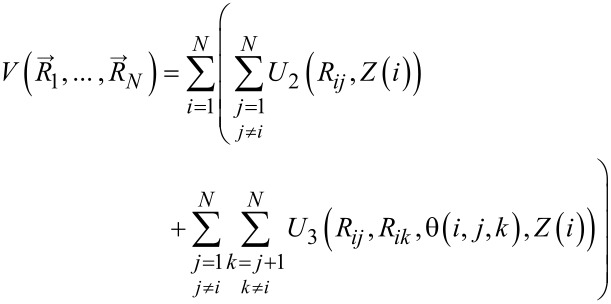


which account for the various binding modes. We would not like to repeat the technical details, which are given in [9], but rather show with two figures how such effective potentials work. Figure 1 shows on the l.h.s. the radial dependence of the two-body potential for various coordinations *Z*(*i*), i.e., various numbers of nearest neighbor atoms. The general trend is that the bond weakens and the minimum shifts to larger distances with coordination. On the r.h.s. of Figure 1 a major ingredient to the three-body term is shown that regulates the bonding angles. As one can see, a single carbon with two neighbors leads to a linear configuration, with three neighbors a 120°-configuration is assumed, and so on.

We tested the potential for several typical sp^2^-bonded materials such as graphene and carbon nanotubes and obtained perfect structures [15]. It should be made clear at this point that classical molecular dynamics cannot describe electronic properties or molecular orbitals, but structure in the sense of atomic positions and mechanical properties such as vibrational spectra or Young’s moduli [16].

## Theoretical method

Our theoretical investigations comprise the generation of initial arrangements of 10 × 10 biphenyl carbon skeletons, i.e., 1200 carbon atoms. The calculation includes only carbon atoms since the hydrogen atoms are removed by irradiation with 50–100 eV electrons at a dose of 50 mC/cm^2^ [4]. Our calculations thus assume that the hydrogens are practically removed at once and not stepwise. It would be an interesting question whether a sequential removal of hydrogen would induce strong correlations of binding sites of neighboring biphenyls [17]. For such an investigation classical potentials for hydrocarbons would have to be used [13–14]. The thiol groups as well as the metallic support are absent in our simulation. Since the initial state is an overall planar configuration the energy minimization always leads to planar configurations even without support.

**Figure 2:**
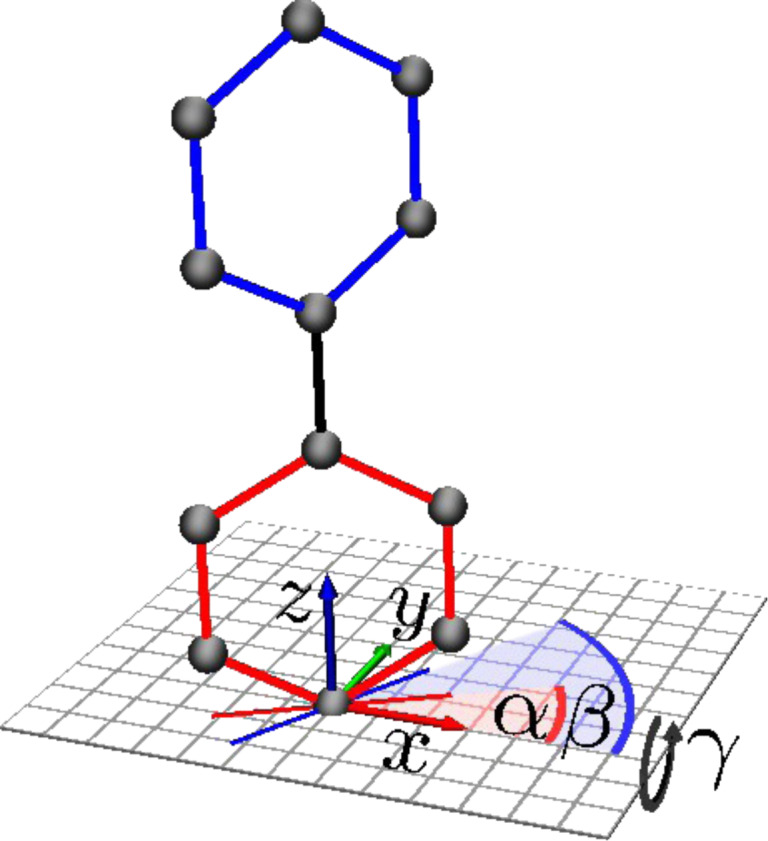
Sketch of the parameters describing a single BPT precursor molecule: α and β denote the rotational angles about the *z*-axis of the lower and upper phenyl relative to the *x*–*y* surface coordinate system (anticlockwise from *x*-axis); their difference gives the twist angle. The tilt angle γ parameterizes the canting of the molecular axis with respect to the surface given by an anticlockwise rotation about the *x*-axis.

For the initial arrangement of the SAM we assumed various geometries. This has two reasons: BPT molecules self-assemble in various structures (hexagonal 2 × 2, herringbone, 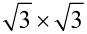) depending for instance on the metallic support [18–21]. But, maybe even more importantly, they rearrange in the process of irradiation in an uncontrolled manner (as they also do at elevated temperatures) [22–23]. Therefore, we investigated the initial arrangements on quadratic, triangular (corresponds to hexagonal 2 × 2), and herringbone lattices. In addition the BPT molecules were collectively oriented according to the angles α 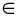 [0°,360°], β 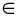 [0°,360°], and γ 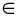 [0°,40°] as depicted in Figure 2. We varied the angles in steps in order to search for dependencies on initial conditions. We also varied α and β independently to allow for dihedral twistings between the upper and lower phenyl rings, that following [4] remain sizable in the SAM in accord with theoretical calculations [6–7].

On top of these initial conditions we also investigated highly excited initial states. These were realized by randomly displacing individual carbon atoms by up to three Ångstroms from their equilibrium position in a phenyl. For larger displacements the respective bonds can be considered as broken. As will be discussed later, our numerical simulations strongly suggest that initial states with broken bonds are the only ones that yield two-dimensionally cross-linked membranes.

After having set up the initial state, we perform an energy minimization by means of the method of steepest descent in order to reach a local energy minimum. Again, as will be discussed later, we are convinced that local energy minima play an important role in the process of membrane formation.

## Results and Discussion

**Figure 3:**
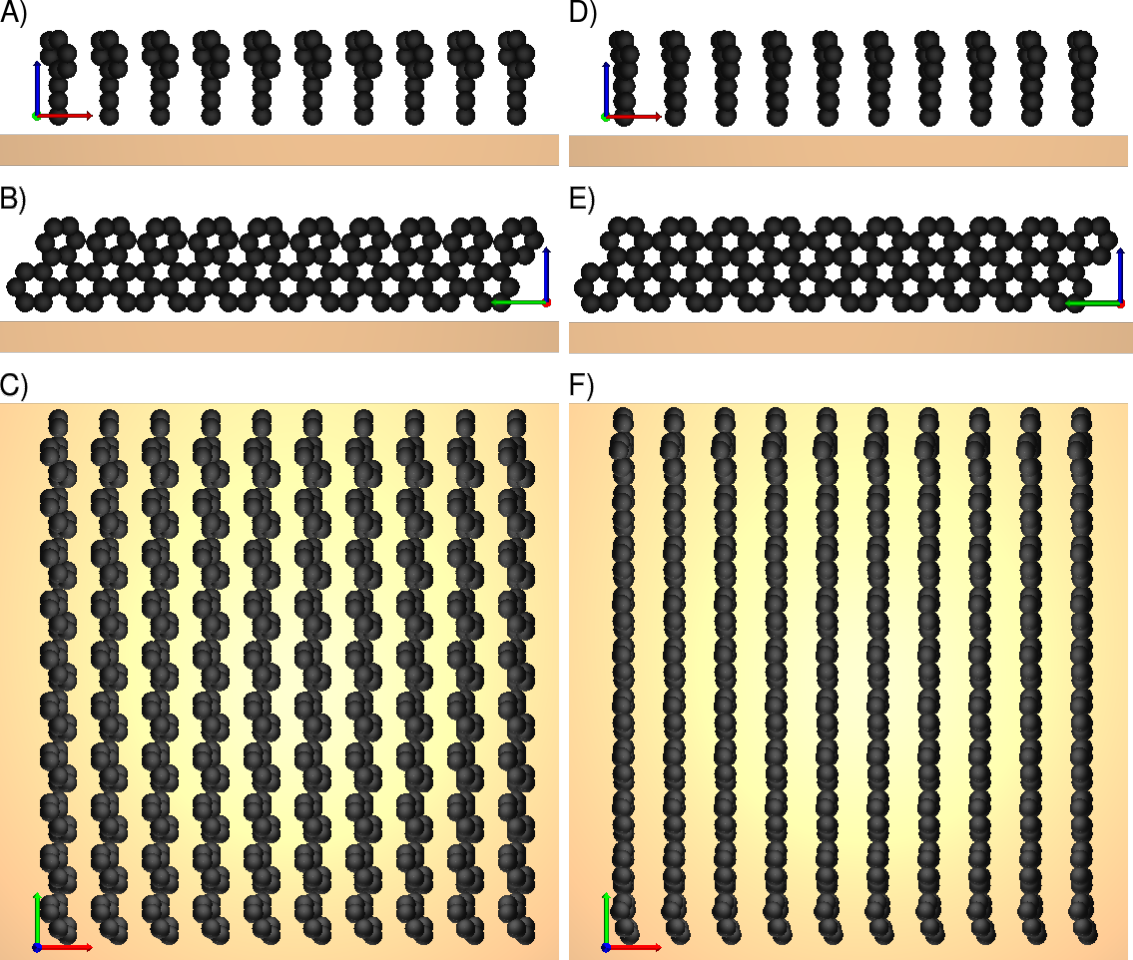
Front (A), side (B) and top (C) view of an initial state made of a regular (square lattice) arrangement of tilted biphenyl carbon skeletons. The tilt angle is γ = 30°. Front (D), side (E) and top (F) view of the resulting local energy minimum state, which consists of regular graphene stripes.

For the discussion it is absolutely essential to understand the following, on first sight not intuitive, concept: If one performs an energy minimization one usually looks for the global energy minimum and does not want to get trapped in a local one. Here it is the opposite. In a unrestricted energy minimization a carbon system would very likely approach a solid such as graphite. If we do not allow the phenyls to break the next better ground state is graphene, and if we do not allow the BPTs to arrange in a common plane, then the ground state is given by graphene like stripes, see Figure 3. These states are not hypothetical or mere products of our classical molecular dynamics calculations: In experiment graphene forms indeed by heating the CNM [4,23], and the stripe order is also found in other calculations [8]. Therefore, we conjecture that the realistic CNM state is described by a local energy minimum, as in other disordered systems like for instance metal glasses [24–26], and this is an important conceptual ingredient of the modeling.

Figure 3 demonstrates how the energy minimum (local and global) looks like if one starts with a regular (square lattice) array of intact biphenyl carbon skeletons. As soon as they acquire some tilt angle they arrange in form of graphene stripes. This is practically independent of the twist angle and the lateral geometry of the SAM. The stripe structure forms even if the initial state is moderately excited by carbon displacements about their mean positions. Interestingly a tilt angle of γ = 31° with respect to the surface normal, which is close to the ideal one of 30°, was determined by means of near-edge X-ray absorption spectroscopy (NEXAFS) investigations on pristine monolayers [1,4,27]. This angle seems to increase, on average, to 41° after irradiation [4]. We speculate that this could mean that some part of the CNM indeed has a graphene-like structure. But as one can easily deduce from Figure 3 such arrangements are interlinked only in one dimension, but not in two.

**Figure 4:**
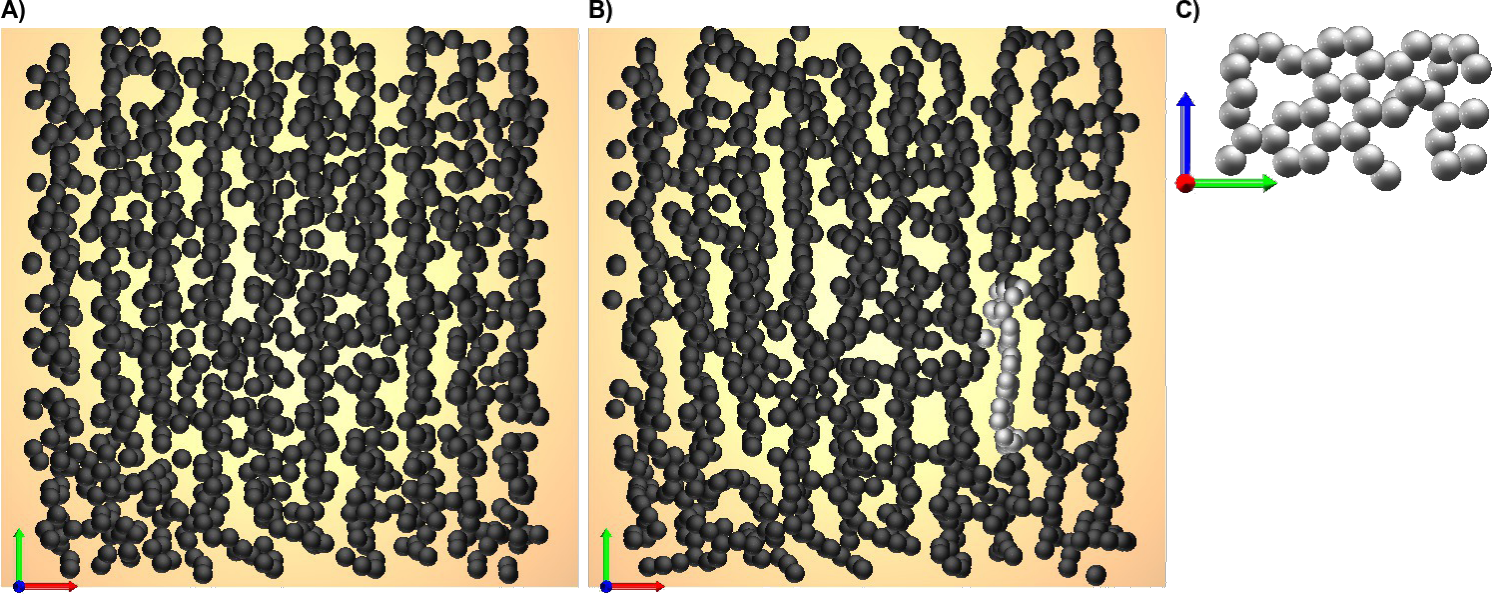
Top view of a strongly excited initial state (A) and of the corresponding local energy minimum state (B). (C) shows the side view of the highlighted region in (B).

We are convinced that the spectroscopically observed amount of destroyed phenyls, [4], plays an important role in understanding the formation of laterally cross-linked biphenyl carbon backbones. It signals that the CNM is very likely laterally interlinked through broken phenyl bonds. Figure 4 shows in panel (A) as an example an initially randomized configuration as it could possibly be realized during the electron exposition and in panel (B) the resulting state (local minimum) found by steepest descent. Panel (C) shows the highlighted region of (B); this structure consists of intact phenyl rings in the middle part that are laterally not cross-linked and of broken phenyl bonds, which provide arms to neighboring parts of the CNM. The amount of broken phenyls varies depending on the excitation of the initial state. As panel (C) of Figure (4) shows, the amount of destroyed rings could easily reach 50%. This is in accord with recent experimental investigations of a loss of aromaticity of terphenylthiol SAMs when being irradiated [17]. Nevertheless, the (remaining) intact phenyl rings seem to play an important role as a kind of matrix that stabilizes the resulting structures [28].

Such structures as depicted in Figure 4 tend to contradict our expectations since they are rather disordered and they contain holes/voids. We do know that the CNMs are mechanically stable, but this does not contradict holes at the nanometer scale. Whether such a porous structure is indeed realized and how such holes are distributed both in size and location could for instance be investigated by means of gas permeation. In addition, the classical picture of point-like particles and voids is somewhat misleading since it does not display the spatial electronic density. In addition the voids suggest that there is no interaction, but the classical carbon–carbon potential acts of course also across voids. Nevertheless, a proper definition of stability remains a challenge.

**Figure 5:**
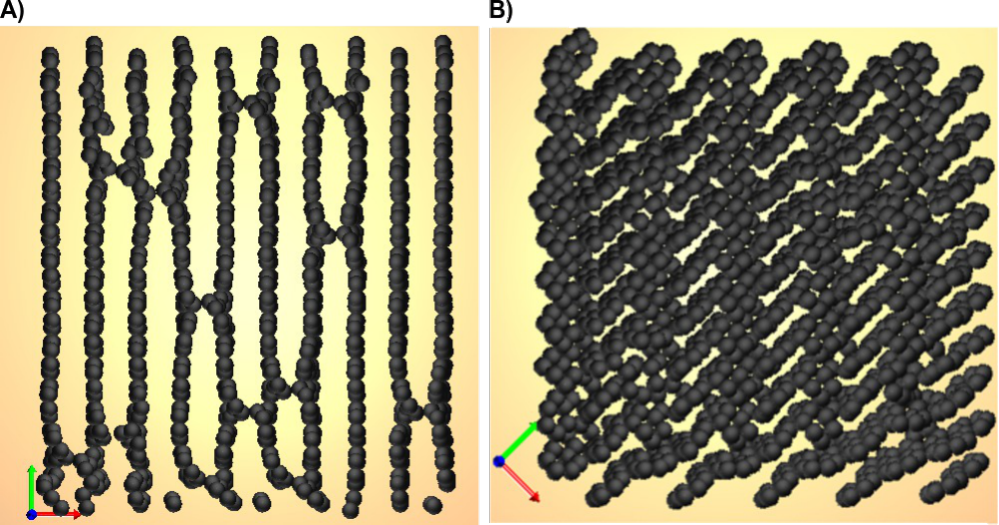
Examples of final structures for an initial triangular lattice geometry (A) and an initial herringbone geometry (B).

In total we performed about 100 simulations starting from square lattice geometries, about 10 for initial herringbone arrangements, and about 60 simulations starting from triangular lattices for various initial conditions characterized by the tilt angle γ, the torsion angles, α and β, of the phenyls, and by the initial displacements of the carbon atoms from their biphenyl positions. Figure 5 shows two more examples of structures that were built in our simulations. Panel (A) shows a final state that is reached on a triangular lattice geometry with moderate initial excitations. It demonstrates once more that systems with intact phenyls tend to form graphene stripes, in this case interlinked by a few broken phenyl bonds. Panel (B) displays a final state starting with a herringbone geometry which looks much denser and almost hole-free.

Although the degrees of freedom chosen in our simulations constitute an enormous parameter space, we are able to summarize our numerical experience like follows: For local displacements of less than about ±1 Å and α not too different from β, the graphene stripe configuration always forms (for all reasonable γ). This is in part also observed in DFT calculations [8]. Only for a sufficient randomization of the initial state, which corresponds to a substantial excitation and to the breakup of sufficiently many phenyls, a true cross-linking is observed. The resulting states are characterized by an irregular structure with pores of various sizes, as can be seen in Figure 4.

We would like to add a final comment on a theoretically possible, very regular arrangement of biphenylthiols, namely a lattice structure where the lower phenyls are interlinked along one direction and the upper phenyls along a different, e.g., orthogonal, direction. This network rests on the special property of biphenyls to allow for a dihedral twist between the two phenyls. But in view of the many very different and in many cases rigid precursors [3] that all form carbon nanomembranes we would like to discard cross-linking mechanisms that rest on very special properties of just one molecule.

## Conclusion

Our investigations demonstrate that carbon nanomembranes, which are produced from molecular precursors such as biphenylthiols, very likely constitute irregular metastable configurations that form from highly excited randomized self-assembled monolayers. This suggests that the electron exposition (dose, time, energy) as well as the cooling dynamics play an important role for the actual structure. To mention just one aspect: In the real crosslinking process hydrogens are removed one by another in the course of time which could induce correlations for possible crosslinking pathways, namely where two hydrogens are removed at nearby sites [17]. Or is this not important and one can start the simulation – as we did – with hydrogen-free initial configurations?

Our investigation is only a first step towards a deeper understanding of the structure and formation of carbon nanomembranes. Future investigations will focus on the dynamical aspects of the membrane formation. For such simulations thermostatted classical molecular dynamics [29–33], with possibly time-dependent temperature profiles have to be used, compare [24–26] for similar attempts in the field of metal glasses. In addition it is an interesting question how the theoretical findings can be compared to experiments. One promising observable is certainly the aromaticity, which can be determined spectroscopically [4,17]. Are STM investigations able to discriminate between the scenarios [3,21,23]? Are gas permeation experiments and bulge tests of Young’s moduli meaningful checks of the structure [16]? And how do our findings transfer to the many other nanomembranes produced from very different precursors?
